# Broadly Active Antiviral Compounds Disturb Zika Virus Progeny Release Rescuing Virus-Induced Toxicity in Brain Organoids

**DOI:** 10.3390/v13010037

**Published:** 2020-12-29

**Authors:** Aleksandra Pettke, Marianna Tampere, Robin Pronk, Olov Wallner, Anna Falk, Ulrika Warpman Berglund, Thomas Helleday, Ali Mirazimi, Marjo-Riitta Puumalainen

**Affiliations:** 1 Science for Life Laboratory, Department of Oncology-Pathology, Karolinska Institutet, 171 65 Stockholm, Sweden; aleksandra.pettke@ki.se (A.P.); marianna.tampere@ki.se (M.T.); olov.wallner@scilifelab.se (O.W.); ulrika.warpmanberglund@scilifelab.se (U.W.B.); thomas.helleday@scilifelab.se (T.H.); 2 National Veterinary Institute, 756 51 Uppsala, Sweden; Ali.Mirazimi@ki.se; 3 Department of Neuroscience, Karolinska Institutet, 171 77 Stockholm, Sweden; pronkrj@gmail.com (R.P.); Anna.Falk@ki.se (A.F.); 4 Unit of Clinical Microbiology, Department of Laboratory Medicine, Karolinska Institute and Karolinska University Hospital, 171 77 Stockholm, Sweden

**Keywords:** Zika virus, pathogenic RNA viruses, antivirals, brain organoids, mode-of-action

## Abstract

RNA viruses have gained plenty of attention during recent outbreaks of Severe acute respiratory syndrome coronavirus 2 (SARS-CoV-2), Zika virus (ZIKV), and Ebola virus. ZIKV is a vector borne Flavivirus that is spread by mosquitoes and it mainly infects neuronal progenitor cells. One hallmark of congenital ZIKV disease is a reduced brain size in fetuses, leading to severe neurological defects. The World Health Organization (WHO) is urging the development of new antiviral treatments against ZIKV, as there are no efficient countermeasures against ZIKV disease. Previously, we presented a new class of host-targeting antivirals active against a number of pathogenic RNA viruses, such as SARS-CoV-2. Here, we show the transfer of the image-based phenotypic antiviral assay to ZIKV-infected brain cells, followed by mechanism-of-action studies and a proof-of-concept study in a three-dimensional (3D) organoid model. The novel antiviral compounds showed a therapeutic window against ZIKV in several cell models and rescued ZIKV-induced neurotoxicity in brain organoids. The compound’s mechanism-of-action was pinpointed to late steps in the virus life cycle, impairing the formation of new virus particles. Collectively, in this study, we expand the antiviral activity of new small molecule inhibitors to a new virus class of Flaviviruses, but also uncover compounds’ mechanism of action, which are important for the further development of antivirals.

## 1. Introduction

The World Health Organization’s (WHO) Blueprint priority diseases indicate pathogens that pose the greatest public health risk due to their epidemic potential and a lack of countermeasures, urging global research and development efforts on these diseases. Zika virus (ZIKV) is one of the priority diseases, along other emerging RNA viruses, such as Severe acute respiratory syndrome coronavirus 2 (SARS-CoV-2) [1]. 

ZIKV is a vector borne Flavivirus that carries its genetic material as single stranded RNA. In 2015, ZIKV emerged in South America, causing a major outbreak in the Americas and it was declared a public health emergency of international concern by WHO in 2016 [2]. In the majority of patients, ZIKV only causes mild diseases with symptoms, including fever, conjunctivitis, headache, and a skin rash. However, ZIKV has been shown to be teratogenic, leading to miscarriages [3] and various neurological complications in newborns after ZIKV infection of pregnant women. The neurological complications have been linked to ZIKV infection of neuronal progenitor cells and the induction of apoptosis and cell death [4,5]. ZIKV-induced neurological cell death in fetuses is the leading cause of microcephaly, a reduction of brain volume, one of the most prominent complications of pregnancy that are associated with ZIKV infection [6]. 

Regarding other Flaviviruses, there is a lack of approved therapies against ZIKV, limiting the treatment options to supportive care. The small genome size of RNA viruses makes them highly dependent on their host cells for replication [7] and makes host-targeting antivirals an attractive approach. Targeting host cell pathways instead of virus proteins directly also facilitates omitting the fast resistance development that RNA viruses face, due to their high mutation rates [8,9]. 

The Helleday laboratory has previously shown that compounds from our in-house library are active as host-targeting antivirals against a broad selection of pathogenic RNA viruses, such as SARS-CoV-2, Ebola virus, and Crimean-Congo hemorrhagic virus [10]. Because the newly identified compound shows antiviral activity across viral genera, the ZIKV pandemic in 2016 prompted us to also test our compound on flavivirus ZIKV. Here, we assessed the antiviral effects and mode-of-action of the newly described compounds in the context of ZIKV infection. The new class of antiviral compounds disturbs ZIKV progeny release and reduces ZIKV-induced toxicity in three-dimensional (3D) brain organoids. Collectively, the new antivirals have broad antiviral activity on a number of emerging RNA viruses, including ZIKV, highlighting their potential for further drug development efforts. 

## 2. Materials and Methods 

### 2.1. Biosafety

Work with infectious viruses were performed under corresponding biosafety laboratory conditions according to the Swedish Work and Health Authorities. Experiments using HAZV and ZIKV were performed under BSL2 conditions.

### 2.2. Cells and Viruses

Human glioblastoma U87-MG (ECACC 89081402) [11] and African Green Monkey Vero (ATCC^®^ CCL-81^™^) cells were grown in complete Dulbecco’s modified Eagle medium (DMEM; Gibco, Waltham, MA, USA) and human adrenal gland carcinoma SW-13 (ATCC^®^ CCL-105^™^) in Leibovitz L-15 medium (Gibco, Waltham, MA, USA) that was supplemented with 10% (*v/v*) Fetal Bovine Serum (FBS, Thermo Fisher Scientific, Waltham, MA, USA), 50 U/mL Penicillin, and 50 μg/mL Streptomycin (Thermo Fisher Scientific, Waltham, MA, USA). U87, Vero, and SW-13 cell lines were maintained at 37 °C under 5% CO_2_, except SW-13 cells, which were maintained under atmospheric CO_2_. Albopictus C6/36 cells were maintained in Eagle’s Minimum Essential Medium (EMEM, Sigma–Adrich, St. Louis, MO, USA) medium that was supplemented with 10% (*v*/*v*) FBS (Thermo Fisher Scientific, Waltham, MA, USA), 1% (*v*/*v*) non-essential amino acids (NEAA, Thermo Fisher Scientific, Waltham, MA, USA) and 1% (*v*/*v*) Sodium Pyruvate (Thermo Fisher Scientific, Waltham, MA, USA) at 28 °C under atmospheric CO_2_. Routinely, Mycoplasma tests were performed while using luminescence-based MycoAlert kit (Lonza, Bazel, Switzerland).

ZIKV (strain H/PF/2013, obtained from European Virus Archive, Marseille, France), stocks were amplified in C6/36 cells. The HAZV (isolate JC280; from European Virus Archive, Marseille, France) stocks were amplified in SW13 cells. Virus titers were determined in Vero cells by end-point dilution assay combined with high-throughput immunofluorescence microscopy of viral protein staining. 

### 2.3. Virus Inoculation and Compound Treatments

The cells were always treated with compounds after virus inoculation, unless otherwise stated. Virus inoculation was performed (MOI 0.1, 0.5, 1, or 10) in 50 μL corresponding cell culture medium for 1 h. At least three wells of cells in a 96-well plate were left uninfected as a mock control. The inoculation medium was removed and replaced with 100 μL dimethyl sulfoxide (DMSO, Sigma–Adrich, St. Louis, MO, USA, max. 0.5% *v*/*v*) or compound-containing medium in at least technical duplicates. The number of infected cells, cytotoxicity, and progeny virus yield in supernatants were determined after 1–3 days post infection (dpi) by end-point dilution assay or virus infectivity assay.

Human brain organoids were inoculated with 2 × 10^4^ ZIKV particles per organoid in the cerebral differentiation medium at development day 14–15 for 24 h. Virus-containing medium was removed and replaced with cerebral differentiation medium containing the desired concentration of compound or equal volume of DMSO (max. 0.1% *v*/*v*). At 3 dpi, the supernatants were collected and replaced with fresh cerebral differentiation medium containing the desired concentration of compound or equal volume of DMSO (max. 0.1% *v*/*v*). At 7 dpi, half of the organoids were collected for downstream assays, and supernatants were collected and replaced with fresh cerebral differentiation medium containing the desired concentration of compound or equal volume of DMSO (max. 0.1% *v*/*v*). At 10 dpi, the remaining half of the organoids were collected for downstream assays.

### 2.4. Phenotypic Antiviral Assay

The screening compounds were spotted on 96-well plates (Falcon^®^ 96-well Black/Clear Flat Bottom TC-treated Imaging Microplate, Corning, Glendale, AZ, USA) in order to reach a final dose of 10 μM (max. DMSO 0.1% *v/v*) and were stored at 4 °C. Prior to performing the experiments, the compounds were thawed at room-temperature, dissolved in media, and distributed on the cells according to the experimental set-up.

HAZV, SW13 cells: 5 × 10^3^ SW13 cells were seeded into 96-well plates and allowed to attach overnight. The cells were inoculated with HAZV in L15 medium in the presence of 10% FBS and 1% PS for 1 h.

ZIKV, U87 cells: 3.5 × 10^3^ U87 cells were seeded into 96-well plates and allowed to attach for 48 h. The cells were inoculated with ZIKV in DMEM medium in the presence of 10% FBS and 1% PS for 1 h.

Virus-containing medium was discarded and replaced with medium containing DMSO (max. 0.5% *v*/*v*) or compounds. The infected cells were incubated in the presence of treatment for 24 or 48 h. Cells were thereby fixed in 4% paraformaldehyde (Santa Cruz, Dallas, TX, USA) in PBS (Gibco, Waltham, MA, USA) for 20 min., followed by the addition of 1:1 (*v*/*v*) methanol-acetone for 20 min. in −20 °C. The number of infected cells was quantified by high-throughput microscopy. Viral titers were quantified by end-point dilution assay using high-throughput microscopy. Data were normalized to DMSO-treated controls.

### 2.5. Determining Antiviral and Cytotoxicity EC_50_

Compound EC_50_ was determined, as described previously [10]. Briefly, compound doses between 50 µM and 1.526 µM were chosen, for Ribavirin (Sigma–Aldrich, St. Louis, MO, USA) doses between 200 μM and 12.5 μM (final concentration; DMSO 0.5% *v*/*v*) were chosen. The compounds were dispersed in deep-well 96-well plates (Biotix, San Diego, CA, USA) while using the D300e Digital Dispenser (Tecan, Männedorf, Switzerland). Prior to performing the experiment, the compounds were thawed in room-temperature and dissolved in culture media.

ZIKV, U87 cells: 3.5 × 10^3^ U87 cells were seeded into 96-well plates and allowed to attach for 24 h. The cells were inoculated with ZIKV in DMEM medium in the presence of 10% FBS and 1% PS for 1 h.

ZIKV, Vero cells: 5 × 10^3^ Vero cells were seeded into 96-well plates and then allowed to attach for 72 h. The cells were inoculated with ZIKV in DMEM medium in the presence of 10% FBS and 1% PS for 1 h.

Virus-containing medium was discarded and replaced with medium containing DMSO or compounds and cells were incubated for 24 and 48 h. The cells were fixed in 4% paraformaldehyde in PBS for 20 min. and in 1:1 (*v/v*) methanol-acetone for 20 min. in −20 °C and then processed for high-throughput microscopy.

### 2.6. Virus Infectivity Assay

The cells were seeded in 96-well plates (Falcon^®^ 96-well Black/Clear Flat Bottom TC-treated Imaging Microplate, Corning, Glendale, AZ, USA) and they were inoculated with virus in 50 μL corresponding cell culture medium for 1 h. Inoculum medium was discarded and 100 μL medium containing compound or DMSO (max. 0.5% *v*/*v*) was added in at least technical duplicates. After 24–72 h post infection (hpi), the supernatants were collected and used in end-point dilution assay. The infected cells were fixed in 4% paraformaldehyde in PBS for 20 min. and in 1:1 (*v*/*v*) methanol-acetone for 20 min. in −20 °C and processed for high-throughput microscopy

### 2.7. End-Point Dilution Assay by Immunofluorescence

In order to quantify viral progeny in supernatants, 5 × 10^3^ Vero cells were seeded in 96-well plates (Falcon^®^ 96-well Black/Clear Flat Bottom TC-treated Imaging Microplate, Corning, Glendale, AZ, USA). On the following day, supernatants containing infectious virus samples were serially diluted on cells by 10-fold and then incubated for 24 h. On the following day, cells were fixed in 4% paraformaldehyde in PBS for 20 min. and in 1:1 (*v*/*v*) methanol-acetone for 20 min. in −20 °C and processed for high-throughput microscopy.

### 2.8. End-Point Dilution Assay by CPE

In order to quantify viral progeny in supernatants, 5 × 10^3^ Vero cells were seeded in 96-well plates (Falcon^®^ 96-well Black/Clear Flat Bottom TC-treated Imaging Microplate, Corning, Glendale, AZ, USA). The following day, virus-containing supernatant samples were serially diluted on cells by 10-fold and then incubated for five days. Cytopathic effects were visually inspected.

### 2.9. High-Throughput Microscopy

High-throughput microscopy following antiviral phenotypic screening, viral infectivity assay, or end-point dilution assay was performed, as previously described [10]. In brief, the cells were incubated with virus-specific primary antibody (see antibody details in Table S2) in 0.2% bovine serum albumin (BSA, Sigma–Aldrich, St. Louis, MO, USA) 0.1% Triton-X in PBS overnight at 4 °C. The cells were washed 3× with PBS and then incubated with secondary antibody (Donkey-anti-mouse Alexa-488; 1:800) and DAPI (1:1000, see details in Table S3) in 0.2% BSA 0.1% Triton-X in PBS for 1 h. The cells were washed 3× with PBS and retained in 100 μL PBS for image acquisition. The images were automatically captured with ImageXpress Micro XLS Widefield High-Content Analysis System (Molecular Devices) using 10× Plan Fluor 0.3 NA objective. The images were quantitatively analyzed by open-source software CellProfiler (Broad Institute, Cambridge, MA, USA) pipeline.

### 2.10. Z-Factor Calculations

The Z-factors were calculated and interpreted, as described previously [12]. Briefly, the threshold for negative controls was defined as the main signal of negative controls plus three times the standard deviation (SD) of those values. Subsequently, the threshold for positive controls was defined as the mean signal of positive controls minus three times the SD of those values. The difference of both thresholds was calculated and divided by the absolute value of the difference of thresholds. Uninfected DMSO-treated cells were used as negative control in infection assays, while the infected DMSO-treated cells were used as positive control. In viability assays, media-only served as negative control and the DMSO-treated cells as positive control.

### 2.11. Generation of Human Cerebral Organoids from hiPS Cells and ZIKV Infection

The human iPS cells were maintained on Biolaminin LN521 (Biolamina, Stockholm, Sweden) in E8 media (Thermo Fisher Scientific, Waltham, MA, USA) and cell passaging was done while using TrypLE Express (Thermo Fisher Scientific, Waltham, MA, USA). Cerebral organoids were generated, as described previously [13,14]. In brief, the iPS cells were cultured in EB media containing E8 (Thermo Fisher Scientific, Waltham, MA, USA) supplemented with 20% KOSR (Thermo Fisher Scientific, Waltham, MA, USA) and 10 µM ROCK inhibitor Y27632 (Millipore, Burlington, MA, USA). Approximately 9000 single iPS cells were plated in EB medium in low adhesive 96-well plates (Corning^®^ Costar^®^ Ultra-Low Attachment Multiple Well Plate; Sigma–Aldrich, St. Louis, MO, USA). Until day 6, half of the media was replaced every second day with EB media without the ROCK inhibitor. Subsequently, the media was switched to neural induction media containing 1:1 DMEM-F12 (Thermo Fisher Scientific, Waltham, MA, USA) and Neural Basal media (Thermo Fisher Scientific, Waltham, MA, USA), N2 supplement (1% *v*/*v*; Thermo Fisher Scientific, Waltham, MA, USA), GlutaMAX supplement (1% *v*/*v*; Thermo Fisher Scientific, Waltham, MA, USA), and MEM-NEAA (1% *v*/*v*; Sigma–Aldrich, St. Louis, MO, USA), PS (1% *v*/*v* Thermo Fisher Scientific, Waltham, MA, USA), heparin (1 µg/mL; Sigma–Aldrich, St. Louis, MO, USA). Until the emergence of clear pseudostratified epithelium, medium was exchanged every second day. Afterwards, aggregates were transferred to growth factor reduced matrigel droplets (VWR, Radnor, PA, USA) in 60 mm culture dishes as described in detail in [13]. Four days of static culture were followed by a change from neural induction medium to cerebral differentiation medium containing 1:1 DMEM-F12 and Neurobasal medium, N2 supplement (0.5% *v*/*v*), insulin (0.25% *v*/*v*; Sigma–Aldrich, St. Louis, MO, USA), GlutaMAX supplement (1% *v*/*v*), MEM-NEAA (0.5% *v*/*v*) and PS (1% *v*/*v*), 2-mercaptoethanol (1:300.000; Gibco, Waltham, MA, USA), and B27 supplement (2% *v*/*v*; Thermo Fisher Scientific, Waltham, MA, USA) without vitamin A. From day 20 onwards, the cerebral differentiation medium was supplemented with 2% B27 supplement with vitamin A (Thermo Fisher Scientific, Waltham, MA, USA) and the organoids were placed on an orbital shaker, rotating at 60 rpm.

Organoids were infected with ZIKV at day 14–15 and, at various time points, disassociated for viability testing or fixed with 4% paraformaldehyde (VWR, Radnor, PA, USA) for 16–24 h. After fixation, the organoids were placed in 30% sucrose (Sigma–Aldrich, St. Louis, MO, USA) and then stored at 4 °C overnight, after which organoids were embedded using Tissue-Tek^®^ O.C.T. Compound, Sakura^®^ Finetek (VWR, Radnor, PA, USA) and then stored at −80 °C until cryo-sectioning.

### 2.12. Immunohistochemistry

Organoids that were embedded in OCT were cryosectioned, placed on Superfrost Plus™ Adhesion Microscope Slides (VWR, Radnor, PA, USA), left to dry at room temperature for 30 min., and then stored at −80 °C until staining. Before the staining procedures, the slides were removed from −80 °C and let to equilibrate at RT for 2 h. A line was drawn around the tissue area with a hydrophobic DAKO pen (Agilent Technologies, Santa Clara, CA, USA). The sections were washed twice with PBS for 10 min., followed by permeabilization and blocking with 3% BSA in PBS + 0.1% Triton-X-100 for 1 h at RT. Blocking solution was replaced with primary antibody (see details in Table S2) solution (3% BSA in PBS +0.1% Triton-X-100, including respective antibody) and then slides were incubated at 4 °C in a humid chamber overnight. The following day, the slides were washed twice with PBS for 10 min., followed by incubation with secondary antibody solution (3% BSA in PBS + 0.1% Triton-X-100 plus respective antibody, as indicated below) for 60 min. at room temperature in the dark. The slides were washed three times with PBS for 10 min., let to dry slightly before mounting with Prolong Gold Antifade Mountant (Thermo Fisher Scientific, Waltham, MA, USA) and capping with coverglass # 1.5 (Menzel Gläser Cover Glasses, VWR, Radnor, PA, USA). The slides were kept in a horizontal position for 48 h before storage at room temperature.

### 2.13. Immunofluorescence Imaging

U87 cells were grown on 12 mm glass coverslips, which were infected with ZIKV (MOI 1) and treated with indicated compounds or DMSO control. The cells were fixed in 4% paraformaldehyde for 20 min. at RT. Cells were permeabilized in 0.5% Triton-X-100 in PBS for 15 min. at RT, followed by 1 h of blocking with a blocking buffer (4% BSA in PBS). Coverslips were subsequently incubated in primary antibody solution (in 0.2% BSA in PBS; see antibody details in Table S2) overnight at 4 °C. The cells were washed 3 × 10 min. in PBS and then incubated in secondary antibody solution (in 0.2% BSA in PBS), together with DAPI (1:1000) for 1 h. The coverslips were mounted in Prolong Gold mounting medium (Thermo Fisher Scientific, Waltham, MA, USA). The images were acquired with a Zeiss LSM 780 microscope while using 40× oil immersion lens and then processed in ImageJ.

### 2.14. RNA Isolation and Real-Time Quantitative PCR

RNA was isolated following the manufacturer’s instructions while using DirectZOL RNA mini kit (Zymo Research, Murphy Ave, Irvine, CA, USA). One-step qRT PCR was performed using TaqMan™ Fast Virus 1-Step Master Mix (Thermo Fisher Scientific, Waltham, MA, USA). For intracellular ZIKV RNA levels, normalization was performed to hbActin expression in uninfected, DMSO treated samples while using ΔΔCT Method. For supernatants, the obtained Ct values were converted to the number of ZIKV RNA molecules using a standard curve that was created using Quantitative Synthetic ZIKV RNA from ATCC (ATCC^®^ VR-3252SD™). Details of the primers used can be found in Table S1.

### 2.15. Cell Viability Assays

The U87 cells were seeded into 96-well plates and then allowed to attach for 72 h. Compounds were dissolved in culture medium with a stable DMSO concentration of maximally 0.5% throughout the plate, added on cells, and the cells were incubated for 24, 48, or 72 h. Thereby, culture medium containing 10 μg/mL Resazurin (Sigma–Aldrich, St. Louis, MO, USA, stock at 1 mg/mL in PBS) was added to cells and then incubated for 4 h. Fluorescence was measured at 544/590 (excitation/emission) using the Hidex Sense 425–301 reader (Hidex, Turku, Finland). The quality of the assay was determined by Z-score while using cell-free wells as negative and wells containing DMSO treated cells as positive controls. The data were normalized to DMSO-treated controls and curve fitting was performed to determine EC_50_ values for cell viability using a sigmoidal, 4PL model in GraphPadPrism.

### 2.16. Organoid Viability Assay

Organoids were dissociated while using the Neural Dissociation kit (Miltenyi Biotec, Bergisch Gladbach, Germany), as described previously [14]. In brief, single organoids were incubated with the kit’s “Mix 1” at 37 °C for 10 min. and resuspended manually several times before “Mix 2” was added and organoids were again resuspended and incubated at 37 °C for 10 min. Organoid suspensions were centrifuged at 300× *g* for 5 min, the dissociation mix was removed, and the cells were resuspended in organoid media according to the developmental day. All of the cells from each organoid were seeded into 96-well plates that were incubated with 10 μg/mL Resazurin (Sigma–Aldrich, St. Louis, MO, USA, stock at 1 mg/mL in PBS) overnight. Absorbance was measured at 570/610 while using the MPP-96 photometer from Biosan and Quant Assay Software.

### 2.17. Time Course and Time-of-Addition Assays

Time course. Three days prior infection, 5 × 10^3^ U87 cells were seeded into 96-well (for viral infectivity assay) and 1.5 × 10^5^ into six-well (for real-time quantitative PCR) plates. The cells were inoculated with ZIKV (MOI 1) in DMEM medium in the presence of 10% FBS and 1% PS for 1 h. Virus-containing medium was removed and replaced with medium containing DMSO or compounds at desired concentration and the cells were incubated for 24, 48, or 72 h. For virus titer measurements, virus-containing supernatants were removed and they were quantified by end-point dilution assay. For quantifying the infected cells, the cells were fixed with 200 μL 4% PFA for 20 min., followed by 200 μL ice-cold methanol-acetone for 20 min. at −20 °C, and they were quantified by viral infectivity assay. For intracellular vRNA, the cells were lysed TRIzol for 10 min. (Thermo Fisher Scientific, Waltham, MA, USA). For extracellular vRNA, the supernatants were collected in TRIzol LS (Thermo Fisher Scientific, Waltham, MA, USA), as recommended by the manufacturer and stored at −80 °C until extraction. vRNA was quantified by real-time quantitative PCR.

Time-of-addition. The U87 cells were seeded into 96-well plates and then allowed to attach for 72 h. For studying viral entry the cells were pre-treated with DMSO or compounds in culture medium for 1 h duration before virus inoculation in order to prime the cells, compounds were then removed and added again within the ZIKV inoculation medium (MOI 10) for additional 1 h duration during viral entry. After 1 h virus inoculation, virus and compound-containing medium was removed, the cells were washed with PBS, and the virus was replicated for 24 h. For the post-inoculation step, cells were inoculated with ZIKV in the culture medium for 1 h. Virus-containing medium was removed and replaced with medium containing DMSO or compounds and the cells were incubated for 24 h. For the budding step, the cells were inoculated with ZIKV in DMEM medium for 1 h. Virus-containing medium was removed and replaced with fresh medium and the cells were incubated for 22 h. After 22 h, medium was removed and replaced with medium containing DMSO or compounds and cells were incubated for 2 h. For all of the treatment strategies, virus-containing supernatants were collected at 24 hpi and used in end-point dilution assay and viral infectivity assay. The infected cells were fixed in 4% paraformaldehyde in PBS for 20 min. and in 1:1 (*v*/*v*) methanol-acetone for 20 min. in −20 °C. The infected cells were quantified by Viral infectivity assay and viral titers were quantified by end-point dilution assay.

### 2.18. Viral Budding Assay

Three days prior infection, 1.5 × 10^5^ U87 cells were seeded into six-well plates in 2 mL culture medium and then allowed to attach for 72 h. The cells were inoculated with ZIKV (MOI 10) in the culture medium for 1 h. Virus-containing medium was removed, replaced with fresh medium, and the cells were incubated for 22 h. Next, the medium was removed, cells were washed twice in 2 mL PBS and replaced with 1 mL medium containing DMSO, 5 μM TH6744, or 10 μM TH6744, and the cells were incubated for 2 h. For extracellular virus quantification, supernatants were collected, and viral titers were quantified by end-point dilution assay. For intracellular viral particles, the medium was removed from the cells and the cells were washed twice in 2 mL PBS. A total of 1 mL culture medium was added on the cells, together with six glass pearls (diameter approximately 1 cm), and the plate was covered with parafilm to avoid spills. The cells were mechanically lysed by moving the plate for 5 min. Cell lysate was collected, centrifuged for 5 min. at 300× *g*, and the cell pellet was additionally homogenized by tissue homogenizer. Cell lysate was centrifuged for 5 min. at 300× *g* and supernatant containing intracellular virus particles was collected. Viral titers were quantified by end-point dilution assay.

### 2.19. Synthesis of Compounds

Detailed synthesis of TH6744 and TH3289 are described in [10] and TH5487 in [15]. The synthesis of TH5464 is described in Supplementary Information. 

### 2.20. Statistical Analysis

Statistical significance was determined while using the GraphPad Prism Software (Version 8.4.0). Two-tailed t-tests, one-way ANOVA, or two-way ANOVA with appropriate follow up tests were used. For each data panel, the tests used are stated in the according figure legend. Unless indicated otherwise, data are presented as mean ± SD from at least *n* = 2 biological replicates. The threshold for defining significance was *p* < 0.05 and the significance levels were defined, as follows: * *p* < 0.05, ** *p* < 0.01, and *** *p* < 0.001. For dose-response-testing, curve fitting was performed in order to determine IC_50_s using a sigmoidal, 4PL model in GraphPadPrism with bottom and top constraints at 0 and 100, respectively. If not indicated otherwise, each image shown is a representative of at least *n* = 2 biological replicates.

### 2.21. Ethics Statement

Ethical permits to work with the donated human skin samples, as described previously [16], was granted prior to start of the project (ethical approval number 2012/208-31/3). Informed consent was granted by donors prior to the sampling of skin samples. 

## 3. Results

### 3.1. TH3289 and TH6744 Shows Antiviral Activity against ZIKV

**Figure 1:**
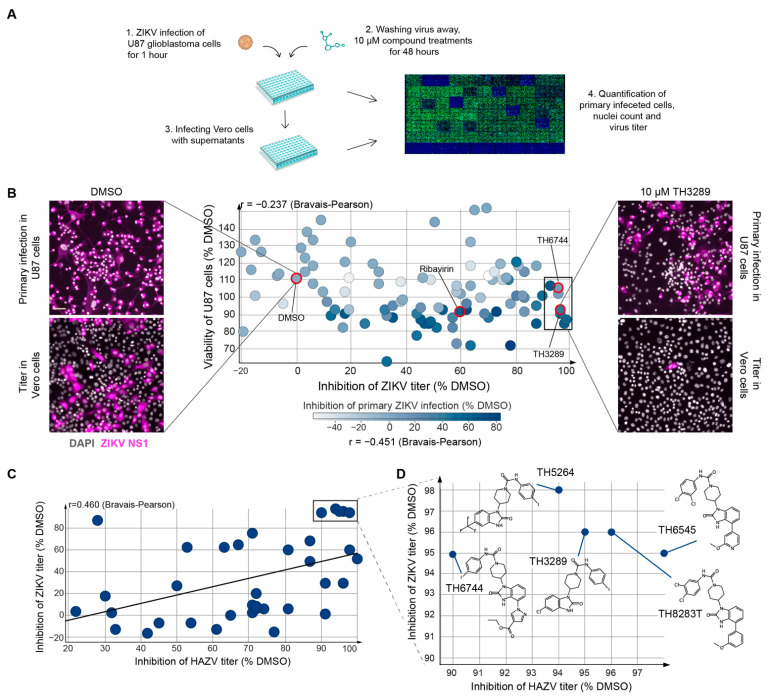
Image-based screening on Zika virus (ZIKV)-infected cells identifies TH3289 and TH6744 as active small molecule inhibitors against ZIKV. (**A**) Experimental workflow of phenotypic antiviral testing of small molecule compounds. Nuclei count was used as an indicator of cytotoxicity, viral antibody staining was quantified to assess the percentage of virus infected cells and virus titer was quantified by end-point dilution assay. (**B**) U87 cells were infected with ZIKV (MOI 10) and treated with structural analogs of TH3289 at 10 µM concentration for 48 h. Infected cells were stained for DAPI and ZIKV NS1 and analyzed by high-throughput imaging. Virus titers from supernatants were determined by end-point dilution assay and inhibition of viral titer was calculated relative to dimethyl sulfoxide (DMSO). Data are presented as a mean of two technical replicates per compound performed in *n* = 1 biological replicate. Representative images from ZIKV-infected U87 cells treated with DMSO or TH3289 (upper image panels; Scale bar equals 100 µm) and corresponding example of titrated ZIKV from the end-point dilution assay (lower image panels) are shown. DAPI in grey, ZIKV NS-1 in magenta. (**C**) The inhibition of viral titers of Hazara virus (HAZV) and ZIKV from both assays was correlated. In the HAZV assay, SW13 cells were infected with HAZV (MOI 10) and treated with 10 μM of compounds from the in-house library for 24 h. Virus titer from supernatant was determined by end-point dilution assay. Cells were stained for DAPI (in blue) and HAZV NP (in green) and analyzed by high-throughput imaging. Data are presented as a mean of two technical replicates per compound performed in *n* = 1 biological replicate. (**D**) Chemical structures of top hits from ZIKV and HAZV antiviral screenings and their respective inhibition on viral titers.

We previously developed an image-based antiviral screening assay while using non-pathogenic Hazara virus (HAZV) as a model virus for RNA viruses [10]. We transferred the assay to ZIKV-infected U87 glioblastoma cells in order to test the antiviral activity of our in-house small molecule inhibitors against a human pathogenic virus (Figure 1A). The antiviral activity of 110 structural analogues of TH3289 was assessed by the quantification of ZIKV-infected U87 cells and progeny release, while compound toxicity was assessed by nuclei count, followed by high-throughput microscopy and automated image analysis while using CellProfiler software (Figure 1A). FDA-approved antiviral Ribavirin was used as a positive control. The treatment of ZIKV-infected cells with 100 µM Ribavirin reduced primary infection by 60% and titer by 63% (Figure 1B). The quality of the antiviral assay was confirmed by a median Z-factor [12] of 0.87 (Figure S1A). From 110 structural analogues of TH3289, the majority had limited effects on ZIKV replication (Figure 1B). Seven compounds reduced primary virus infection by 11–54% and viral titer by >90% without inducing cytotoxicity (cell viability >80%) (Figure 1B, rectangle, Data S1). In agreement with the findings from screening in HAZV [10], TH3289 and TH6744 were confirmed among the top hits (Figure 1B). Additionally, a positive correlation between the antiviral activity of compounds on both ZIKV and HAZV was detected, as illustrated by a Bravais–Pearson correlation score of 0.460 (Figure 1C), confirming the robustness of the assay and antiviral activity of compounds against viruses from two different families. Furthermore, the top five compounds with similar chemical structures resulted in 1-log inhibition on viral titer in both HAZV-infected SW13 cells and ZIKV-infected U87 cells, which suggested that the compound series has antiviral activity on different RNA viruses across cell types (Figure 1C, rectangle and Figure 1D). The top five compounds are benzimidazolones that are substituted with a piperidine ring carrying an aromatic urea moiety and carrying either a small substituent on position 5 or a bulky substituent on position 4 (Figure 1D). Of the top five compounds, TH3289 and TH5264 form a group of very close analogues, as well as TH6744, TH6545, and TH8283T. From each group of chemically strongly related compounds, one or two were chosen for further validation.

### 3.2. Novel Antiviral Compounds Have a Similar Therapeutic Window as Ribavirin against ZIKV in Cellular Models

**Figure 2:**
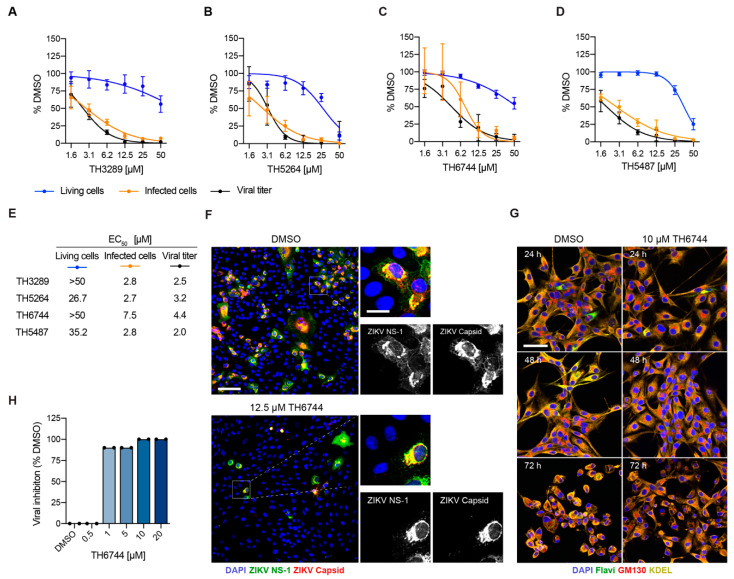
Series of antiviral compounds have a therapeutic window and rescue ZIKV-induced CPE in cellular models. (**A**–**E**) U87 cells were infected with ZIKV (MOI 1) and treated with increasing doses of (**A**) TH3289, (**B**) TH5264, (**C**) TH6744, or (**D**) TH5487 for 48 h. Cell viability was determined by nuclei count (in blue), infected cells by ZIKV NS1 staining (in orange) and virus titer by an end-point dilution assay (in black). Data are presented as mean ± SD from *n* = 3 independent replicates. (**E**) Curve fitting was performed to calculate EC_50_ values for living cells, infected cells and viral titer. (**F**) Vero cells were infected with ZIKV (MOI 10) and treated with 12.5 μM TH6744 or DMSO for 48 h. Cells were stained for ZIKV NS1 (in green), ZIKV Capsid (in red) and DAPI (in blue). Images of *n* = 1 biological replicate. In the overview image, scale bar equals 100 μm and in the close-up 25 μm. (**G**) U87 cells were infected with ZIKV (MOI 1) and treated with 10 μM TH6744 or DMSO for 24, 48, or 72 h. Cells were stained for Pan-Flavi (in green), Golgi marker GM130 (in red), ER marker KDEL (in yellow), and DAPI (in blue). Images of *n* = 1 biological replicate. Scale bar equals 50 μm. (**H**) Vero cells were infected with ZIKV (MOI 0.1) and treated with increasing doses of TH6744 for 24 h. Viral titer was measured from the supernatants by end-point dilution assay on Vero cells and the development of CPE were monitored by visual assessment. Data presented as mean ± SD from *n* = 2 biological replicates.

The EC_50_ values for three of the hits named TH3289, TH5264, and TH6744 were determined in a dose-response treatment in ZIKV-infected U87 cells in order to examine the therapeutic window of the screening hits (Figure 2A–E and Figure S2A, Data S2). Additionally, compound TH5487, a close analogue of the screening hits that has anti-inflammatory properties [15], was included in the dose-response assay in ZIKV-infected U87 cells (Figure 2D, Data S2). TH5487 has been extensively characterized for its metabolic, toxicological, and pharmacokinetic properties [14], providing a good starting point for further compound development, and further supporting inclusion in the antiviral studies. The antiviral effect was confirmed for all four compounds, with EC_50_ values ranging between 2.7 and 7.5 μM on primary virus infection and 2.0 and 4.4 μM on viral progeny release in U87 cells (Figure 2A–E and Figure S2A). A dose-response study in ZIKV-infected Vero cells further confirmed the antiviral therapeutic window of TH6744 (Figure S2B). TH6744 and TH5487 had more favorable toxicity profiles than TH3289 and TH5264 (Figure S2C–G, Data S2), with IC_50_ values >50 μM at all of the measured time points (Figure S2G). A comparison of TH6744 and TH5487 to Ribavirin revealed a similar therapeutic window in ZIKV-infected U87 and Vero cells (Figure S2H,I, Data S2), but both TH5487 and TH6744 were more potent in inhibiting both primary infection and progeny release. 

Compound TH6744 was further used in complementary assays in order to confirm our findings. Stainings of ZIKV NS1 and Capsid proteins were compared to exclude an antibody-based bias in our image-based assay, and they showed the same sensitivity for the detection of ZIKV-infected cells (Figure 2F). In agreement with previous studies, ZIKV infection induced cytopathic effects (CPE) in U87 at 72 h post infection (hpi) (Figure 2G) [17] and in Vero cells at five dpi (Figure 2H). The treatment with TH6744 reversed ZIKV-induced CPE in U87 cells (Figure 2G), as illustrated by the phenotype change of ZIKV-infected U87 cells after TH6744 treatment. Additionally, TH6744 treatment reduced viral titer in a dose-dependent manner in Vero cells measured by CPEs (Figure 2H), confirming the compound’s antiviral activity on ZIKV progeny release while using a secondary readout widely used in the field. Altogether, a dose-dependent decrease of ZIKV-infected cells and viral titer at non-toxic concentrations was observed for both TH6744 and TH5487, confirming their antiviral activity. Based on the antiviral and toxicity data, TH6744 and TH5487 were selected for further studies. 

### 3.3. TH6744 and TH5487 Treatment Reverses ZIKV-Induced Neurotoxicity and Limits ZIKV Propagation in a 3D Cerebral Organoid Model

We first established a ZIKV infection model using cerebral organoids that were derived from induced pluripotent stem (iPS) cells in order to study the antiviral activity of TH6744 and TH5487 in an advanced 3D model, as previously described [5,13,18]. Fourteen days old organoids were infected with increasing ZIKV doses between 1 × 10^3^ and 1 × 10^5^ particles per organoid. ZIKV replication reduced organoid viability by 50% at an infection dose of 1 × 10^3^ particles per organoid and between 10–20% at higher ZIKV doses (Figure S3A). Furthermore, ZIKV infection induced a prominent change in organoid structure (Figure S3B). The detection of ZIKV NS1 protein within organoid sections (Figure S3B) in addition to a dose- and time-dependent increase of viral titer over seven days indicated active ZIKV replication in organoids (Figure S3C).

**Figure 3:**
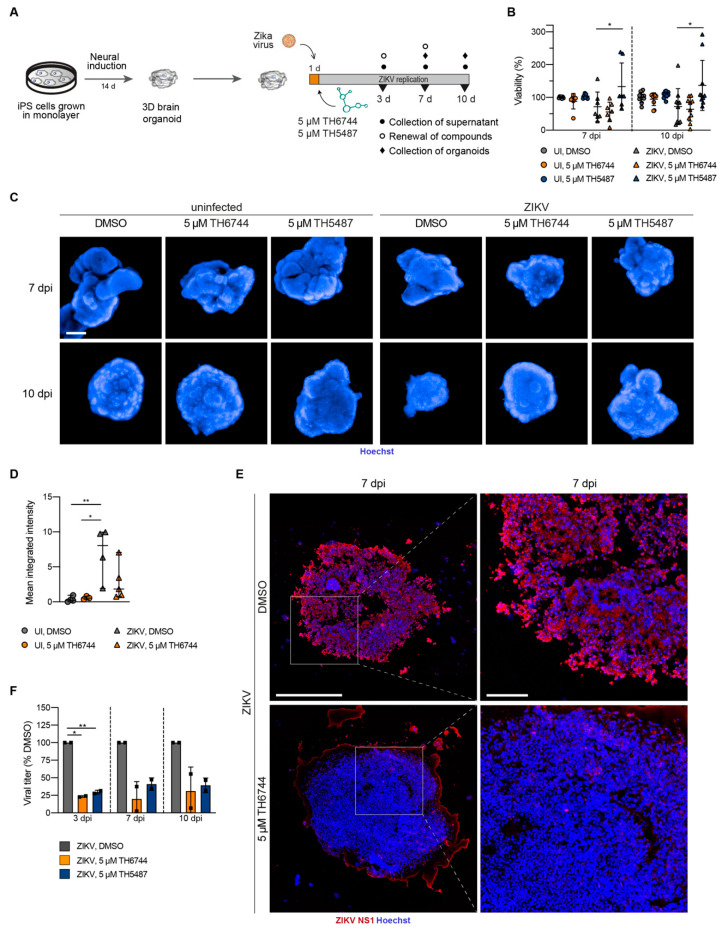
TH6744 and TH5487 treatment reverses ZIKV-induced neurotoxicity and limits ZIKV propagation in three-dimensional (3D) cerebral organoids. (**A**) Experimental workflow of ZIKV infection on brain organoid model. (**B**–**F**) Organoids were infected with ZIKV (2 × 10^4^ particles/organoid) followed by treatment with 5 μM TH6744, 5 μM TH5487 or DMSO. (**B**) Organoids were dissociated to single cells at 7 or 10 dpi and cell viability was measured by Resazurin assay. Viability is shown relative to uninfected DMSO-treated control at respective timepoints. One datapoint represents one organoid. Data are presented as a mean ± SD of *n* = 2 biological replicates. Statistical significance was determined using two-way ANOVA with Dunnett’s multiple comparison analysis. * *p* < 0.05, ** *p* < 0.01, (**C**) ZIKV-infected and uninfected whole organoids treated with TH6744 or DMSO for 7 or 10 dpi, stained by Hoechst and imaged by high-content confocal microscopy. Representative images of *n* = 2 biological replicates per condition. The scale bar equals 500 μm. Hoechst in blue. (**D**) ZIKV-infected organoids that were treated with TH6744 or DMSO were fixed at 7 dpi, cryosectioned and stained with Hoechst and ZIKV NS1 protein antibody. ZIKV NS1 intensity was quantified using Cellprofiler software. Data are presented as single values and mean ± SD from *n* = 2 biological replicates. Statistical significance was determined using one-way ANOVA with Dunnett’s multiple comparison analysis. * *p* < 0.05, ** *p* < 0.01. (**E**) ZIKV-infected organoids treated with TH6744 or DMSO were fixed at 7 dpi, cryosectioned, stained with Hoechst and ZIKV NS1 protein antibody and imaged by confocal microscopy. Representative images of *n* = 2 biological replicates per condition. Scale bar equals 500 μm in overview image and 100 μm in close-up. ZIKV NS1 protein in red, Hoechst in blue. (**F**) Viral titer from ZIKV-infected and compound treated organoids at indicated time points was determined by an end-point dilution assay. Viral titer is presented as relative to ZIKV-infected DMSO-treated control at respective timepoints. Data are presented as a mean ± SD from *n* = 2 biological replicates. Statistical significance was determined using two-way ANOVA with Sidak’s multiple comparison analysis. * *p* < 0.05, ** *p* < 0.01.

Next, we studied the effects of TH6744 and TH5487 on cerebral organoids that were infected with an intermediate virus dose of 2 × 10^4^ ZIKV particles per organoid (Figure 3A). The treatment with TH5487 led to an 35% increased viability as compared to DMSO control at both 7 and 10 dpi (Figure 3B). TH6744 and TH5487 treatment both led to a phenotypic rescue characterized by retained structure of organoids at 7 dpi and 10 dpi (Figure 3C). In organoids that are treated with TH6744, a pronounced reduction in primary virus infection was observed, as depicted by a marked reduction of ZIKV NS1 intensity in sectioned organoids at 7 dpi (Figure 3D,E and Figure S4A,B). Moreover, treatment with both TH6744 and TH5487 reduced ZIKV progeny particles that were produced by the organoids by 70–80% at all timepoints for TH6744 and 60–70% at all timepoints for TH5487 (Figure 3F). Altogether, these findings provide a strong proof-of-concept for compounds’ antiviral activity in a 3D-organoid model.

### 3.4. TH6744 Disturbs Late Steps in ZIKV Life Cycle

**Figure 4:**
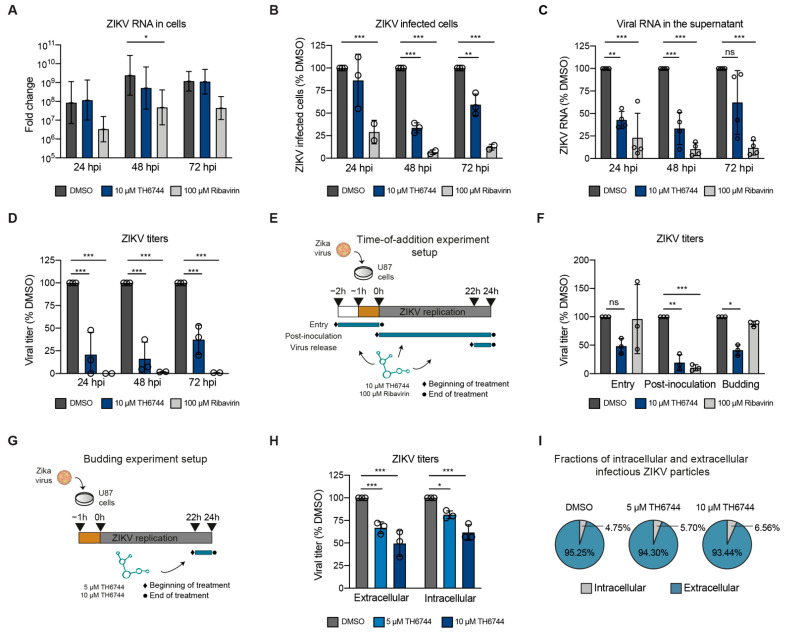
TH6744 disturbs late replication cycle steps in ZIKV life cycle. (**A**–**D**) U87 cells were infected with ZIKV (MOI 1) and treated with indicated compounds for 24, 48 or 72 h. (**A**) ZIKV RNA levels inside the cells were quantified by qPCR. (**B**) Number of ZIKV-infected cells was quantified by virus infectivity assay. (**C**) ZIKV RNA levels in the supernatant were quantified by one-step qRT-PCR. (**D**) ZIKV titers were quantified by end-point dilution assay. (**E**) Schematic overview of time-of-addition experimental setup in U87 cells. (**F**) U87 cells were infected with ZIKV (MOI 10) for a total of 24 h and treated with DMSO control, 10 μM TH6744 or 100 μM Ribavirin for indicated periods during early (entry), late (budding) or throughout replication cycle (post-inoculation). ZIKV titers were quantified by end-point dilution assay. (**G**) Schematic overview of budding experimental setup. (**H**) U87 cells were infected with ZIKV (MOI 10) for 22 h and treated with indicated TH6744 doses for 2 h. Intracellular ZIKV particles were obtained by mechanical cell lysis and extracellular ZIKV particles from the supernatants and both were quantified by end-point dilution assay. (**I**) Average fractions of intracellular and extracellular infectious ZIKV particles of the total particles quantified in (**H**). (**A**–**D**,**F**,**H**) Data are expressed as a mean ± SD from at least *n* = 3 biological replicates. Statistical significance was determined by using one-way ANOVA with Dunnett’s multiple comparison analysis. * *p* < 0.05, ** *p* < 0.01, *** *p* < 0.001.

We recently characterized the cellular pathway-level responses to TH6744 treatment and uncovered a broad response from the Heat Shock Protein 70 (Hsp70) network. In order to study how TH6744 influences virus replication, the ZIKV replication cycle was studied in detail. ZIKV genome replication and progeny release kinetics were studied in U87 cells over three days by qPCR and viral titer measurements, respectively, in order to evaluate the effects of TH6744 on ZIKV kinetics (Figure S5A). At 24 hpi, no reduction of intracellular ZIKV vRNA (Figure 4A) or ZIKV-infected cells (Figure 4B) were detected upon TH6744 treatment without affecting the viability (Figure S5B). However, extracellular vRNA levels and viral titers reduced by 50% and 75%, respectively (Figure 4C,D). At 48 hpi, the intracellular ZIKV vRNA levels remained unchanged by TH6744 treatment (Figure 4A), but the number of ZIKV-infected cells was reduced by 65% (Figure 4B) and titer by 83% (Figure 4D). After 72 h exposure, TH6744 treatment decreased the number of ZIKV-infected cells by 40% (Figure 4B) and viral titer by 63% (Figure 4D), which suggested that TH6744 may partially lose its activity after 72 h possibly due to cellular metabolism of TH6744 and/or compound’s reduced stability in culture medium. Ribavirin reduced intracellular vRNA levels at all timepoints (Figure 4A) and extracellular vRNA and ZIKV titers from 24 hpi and onwards (Figure 4C,D), as expected from a nucleoside analogue. These data illustrate how TH6744 treatment reduces ZIKV progeny virus release during early infection time points, potentially at the first replication round [17], leading to reduction of ZIKV-infected cells during secondary infections while not affecting ZIKV genome replication. 

A time-of-addition study was performed in U87 cells in order to further understand which virus life cycle step is influenced by TH6744 (Figure 4E). When the cells were pre-treated with TH6744 for 1 h and at viral entry during viral inoculation and thereby allowed to replicate for 24 h, a 50% reduction on viral titer was observed, while Ribavirin had no effect on the entry steps (Figure 4F and Figure S5C). When adding TH6744 directly after virus inoculation for 24 h (post-inoculation), a pronounced reduction of viral titer was observed (Figure 4F). Interestingly, when TH6744 treatment was initiated at 22 h after inoculation only for 2 h (virus budding), 50% titer reduction was seen (Figure 4F). Ribavirin treatment, on the other hand, reduced ZIKV titers by 80% at post-inoculation step only (Figure 4F), as expected from a nucleoside analogue, while it had no effect on the later steps. In order to study the budding of virus particles in detail, virus particles from inside (pre-budding) and outside of cells (post-budding) were quantified [19] after 2 h TH6744 treatment (Figure 4G). Interestingly, the infectivity of both pre-budding and post-budding particles reduced to a similar extent upon TH6744 treatment in a dose-dependent manner (Figure 4H) and the fraction of intracellular infectious particles as compared to extracellular remained largely unchanged (Figure 4I), showing no clear influence on the budding efficiency. These results indicate a rapid effect of TH6744 on ZIKV particle traffic into and out of the cells, especially during the late life cycle steps after RNA replication.

## 4. Discussion

In the light of the increasing outbreaks of RNA viruses and Flaviviruses in particular, there is an urgent pressure to develop new, broadly active antivirals. While using a cell-based phenotypic approach, we previously identified a series of small molecule inhibitors with a broad range antiviral activity against emerging RNA viruses [10]. Here, we switched from using HAZV-infected SW13 cells to ZIKV-infected U87 cells and screened a set of compounds, generating the same top-hits. The successful transfer of the screening cascade from HAZV to ZIKV underscores the versatility and robustness of the image-based screening assay. Its transferring capacity across virus families and cell types can help to rapidly develop antiviral screening strategies for future virus outbreaks. 

Phenotypic approaches are dependent on the model of choice and they require thorough validation of hit compounds. While using cell lines in a phenotypic assay is the most feasible, especially in a biosecurity setting, a cell line cannot depict the complexity of an organ, like the human brain. iPS cell derived cerebral organoids are promising tools for modeling typical features of ZIKV infection, like microcephaly and the disruption of cortical development [5]. ZIKV has been shown to target neuronal progenitors in cerebral organoids and, consequently, lead to impaired organoid growth and viability [20,21]. In concordance with these findings, we see a reduced viability and pronounced cell death in organoids upon ZIKV infection, proving them to be a biologically relevant model for studying ZIKV infection. A drawback of this ZIKV 3D infection model is the previously reported size variability between organoids [13], which was evident in the evaluation of organoid viability and made the quantification of organoid size upon infection and treatment over time difficult without following single organoids. Despite the variability in size of the organoids, we show a clear rescue of ZIKV-induced neurotoxicity and reduced viral load in both infected organoids and viral progeny production by treatment with TH6744 and TH5487, providing a proof-of-concept for antiviral compounds in a 3D disease model. However, additional studies are needed in order to determine the cellular composition and, especially, differential infection profiles of the cerebral organoids, as well as investigate differential cell death in infected or non-infected cells within the organoids.

Studying kinetics and time-of-addition are well-established approaches for gaining insights regarding antiviral compound’s mode-of-action, especially when little is known about the molecular target of the compound [22]. When monitoring ZIKV kinetics, we observed that TH6744 does not impair ZIKV vRNA synthesis, but strongly reduces progeny release, in contrast to Ribavirin, which reduces ZIKV vRNA synthesis, as reported previously [23,24,25]. Rapid and significant reduction of ZIKV progeny release by TH6744 treatment, even when administered late in infection for only 2 h, indicates disturbances of ZIKV life cycle at late stages. However, we observed no clear effect on the budding efficiency by TH6744 on pre-budding and post-budding infectious particles. An increase of intracellular infectious particles that are unable to exit the cells would suggest compound inhibiting budding efficiency [26]. Instead, TH6744 reduces both intra- and extracellular infectious particles to a similar extent, suggesting that TH6744 disturbs the ZIKV life cycle after RNA replication, disturbing either viral protein synthesis, post-processing or stability, virion assembly, trafficking, or budding. Therefore, TH6744 proves to not only mitigate ZIKV-induced pathogenic phenotypes, but also interrupt virus transmission. Future multi-step kinetics studies while using low ZIKV doses quantifying intra- and extracellular infectious ZIKV progeny are necessary for further understanding TH6744 activity in more physiological viral doses. Additionally, further cryo-electron microscopy studies on infected cells are needed in order to pinpoint and disseminate at which exact stage TH6744 disturbs ZIKV replication cycle.

We previously characterized the cellular pathway-level responses to TH6744 treatment by implementing mass-spectrometry based target identification method [10]. There, we showed how TH6744 treatment affected multiple components of the host proteostasis pathways, especially members from the Hsp70 chaperone network. Additionally, TH6744 disturbed the interaction between HAZV nucleoprotein and cellular Hsp70 [10]. Several studies have exemplified the importance of HSP70 family members for ZIKV and DENV infection [27,28,29]. In fact, Flaviviruses are highly dependent on a subset of host chaperones for supporting viral replication and protein folding [30], while host cells distribute their needs over a more diverse range of pathways, which creates an opportunity for pharmacological intervention. Given the Hsp70’s crucial role for ZIKV particle production and virion assembly, TH6744 immediate activity on late ZIKV replication cycle presented in the current study, and TH6744 disturbing Hsp70 and viral protein interactions, we propose that TH6744 treatment interrupts the capacity of Hsp70 in order to fold viral proteins and, thereby, leads to the formation of dysfunctional ZIKV particles in human cells and organoid models. Studying the activity of compounds in cells with genetically modified Hsp70 protein levels would unravel the specific effects of TH6744 via disturbing the Hsp70 network in the context of a ZIKV replication cycle.

## 5. Conclusions

In conclusion, this study describes an image-based antiviral screening cascade on ZIKV-infected U87 cells, leading to the discovery of TH6744 and TH3289 as antiviral compounds against ZIKV. The validation by a dose-response assay reveals a favorable toxicity profile of the series of compounds and a therapeutic window that is comparable to FDA-approved antiviral Ribavirin. Alongside antiviral activity, TH6744 and its close analogue, TH5487, improved the viability of infected 3D organoids, illustrating the phenotypic rescue accompanied by the antiviral activity. Importantly, the TH6744 mode of antiviral action was narrowed to ZIKV late replication cycle steps and proposed to be mediated by TH6744 effects on the host Hsp70 pathway.

## Data Availability

The data that support the findings of this study are within the article, supporting information, supplementary data tables, or available from the corresponding author upon request.

## Supplementary Materials

The following are available online at https://www.mdpi.com/1999-4915/13/1/37/s1, Data S1; Data S2; Supplementary Information: Figure S1: Image-based screening quality controls, Figure S2: Toxicity profile and antiviral activity of the compound series and Ribavirin, Figure S3: Establishing a human iPS-cell derived cerebral organoid model to study antiviral activity of TH6744, Figure S4: TH6744 has an antiviral effect in human iPS-cell derived cerebral organoid model, Figure S5: Studying ZIKV kinetics and TH6744 time-of-addition. Supplementary Material and methods: Synthesis of N-(4-iodophenyl)-4-[2-oxo-5-(trifluoromethyl)-2,3-dihydro-1H-1,3-benzodiazol-1-yl]piperidine-1-carboxamide TH5264, Table S1: qPCR primers, Table S2: Antibodies, Table S3: DNA Dyes, Supplementary reference
