# Supplementary material for: Broadly Active Antiviral Compounds Disturb Zika Virus Progeny Release Rescuing Virus-Induced Toxicity in Brain Organoids
Source: Viruses. 2020 Dec 29;13(1):37. doi: 10.3390/v13010037 (PMC7823652; doi:10.3390/v13010037)
Supplement: Supplementary file 1 [file viruses-13-00037-s001.zip › Viruses_Supplementary Material/Pettke et al._Viruses_Supplementary information.pdf]

Supplementary Information

Broadly active antiviral compounds disturb Zika virus progeny release rescuing virus-induced toxicity in brain organoids

Pettke et al.

This file includes:
Figures S1 - S5
Supplementary Materials and Methods
Tables S1 to S3
Supplementary reference

Figure S1. Image-based screening quality controls.

a

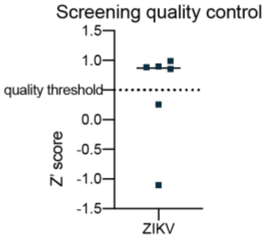

(**A**) Z-factors were calculated from each experiment. Data is presented as median z-factor and single values of six ZIKV assays.

Figure S2. Toxicity profile and antiviral activity of the compound series and Ribavirin.

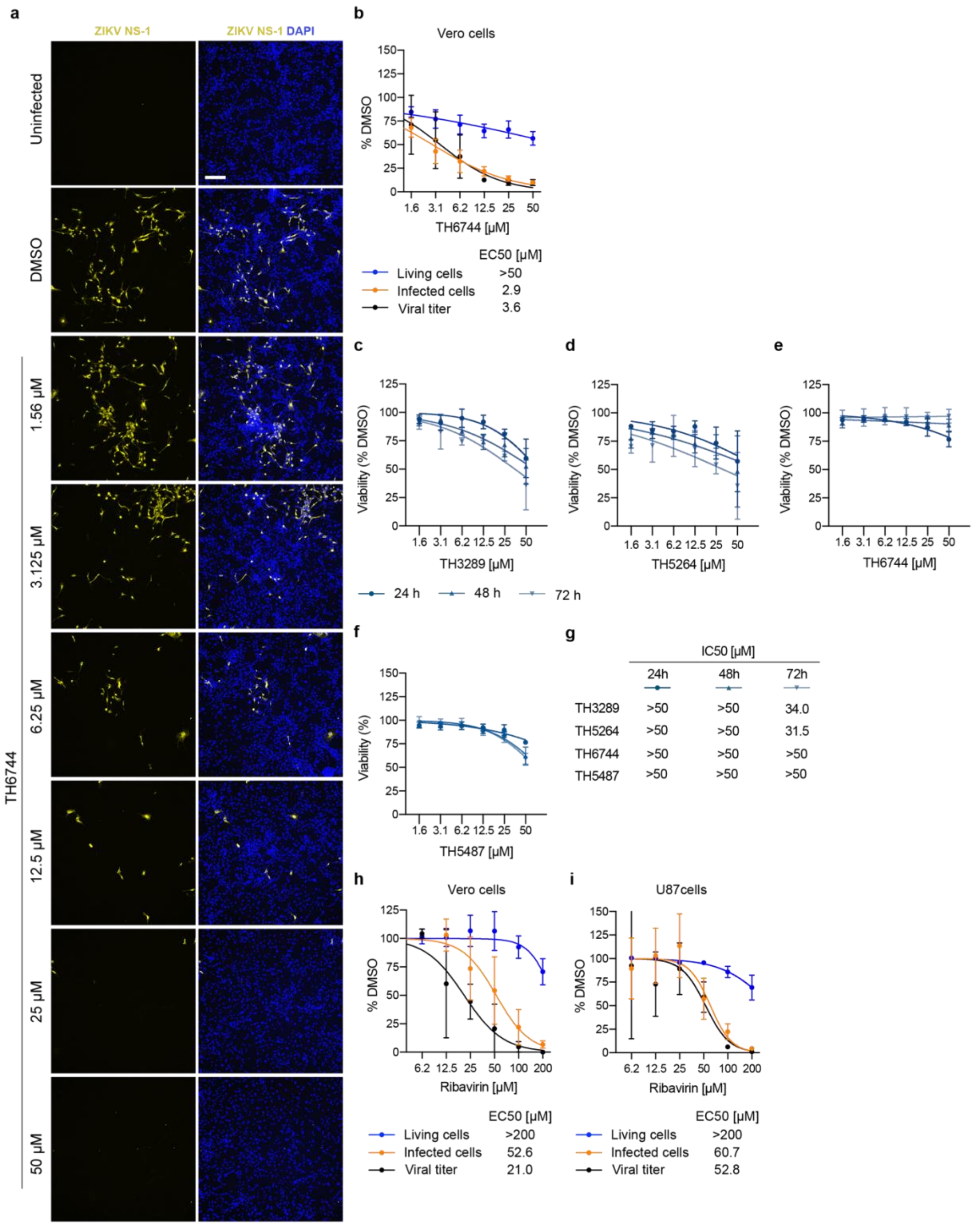

(A) U87 cells were infected with ZIKV (MOI 1) and treated with indicated TH6744 doses for 48 h and processed for IF. Stainings ZIKV NS-1 in yellow and DAPI in blue. Scale bar equals 200 µm. Data is

representative of at least n=3 biological replicates. (**B**) Vero cells were infected with ZIKV (MOI 1) and treated with indicated doses of TH6744 for 48 h. Cell viability was determined by nuclei count (in blue), infected cells by ZIKV NS1 staining (in orange) and virus titer by end-point dilution assay (in black). Data is presented as Mean ± SD from *n*=3 biological replicates. (**C-G**) U87 cells were treated with indicated doses of TH3289 (C), TH5264 (D) TH6744 (D) or TH5487 (F) for 24, 48 or 72 h. Cellular viability was measured by Resazurin assay. Data is normalized to DMSO control and presented as Mean ± SD from n=3 biological replicates. (**H-I**) Vero (H) or U87 cells (I) were infected with ZIKV (MOI 1) and treated with indicated doses of Ribavirin for 48 h. Cell viability was determined by nuclei count (in blue), infected cells by ZIKV NS1 staining (in orange) and virus titer by end-point dilution assay (in black). Data is presented as Mean ± SD from n=2 biological replicates. (**B-I**) Curve fitting was performed to determine EC50s.

Figure S3. Establishing a human iPS-cell derived cerebral organoid model to study antiviral activity of TH6744.

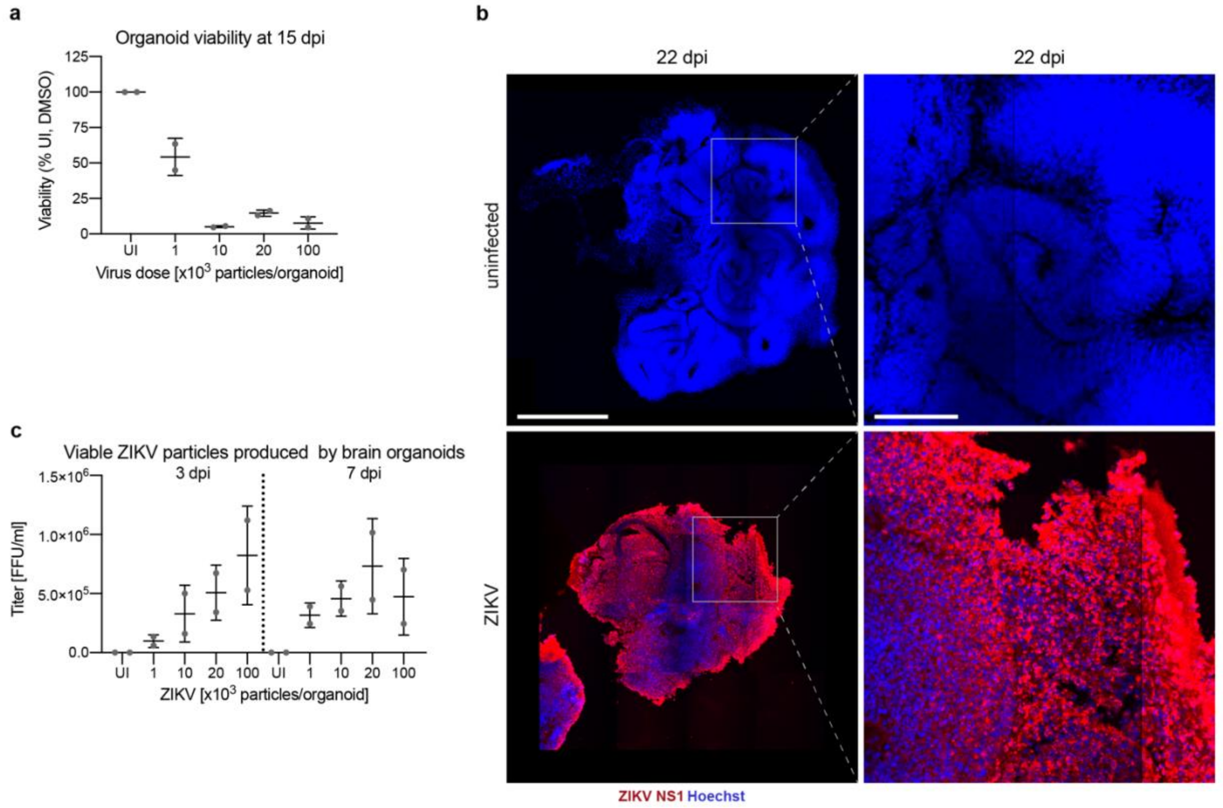

(A-C) 14-day-old human iPS-cell derived organoids were infected with ZIKV doses between 1,000 and 100,000 ZIKV particles per organoid. **(A)** At 15 dpi, organoids were dissociated, and cell viability was determined. One datapoint represents one organoid. Data is presented as Mean ± SD of two technical repetitions per datapoint of n=1 biological replicate **(B)** Organoids were fixed with 4% PFA at 22 dpi, cryo-sectioned and stained for Hoechst and ZIKV NS1 protein. Images were acquired by confocal microscopy. Images of n=1 biological replicate per condition. Scale bars equals 500 µm in overview image, 100 µm in close-up. ZIKV NS1 protein in red, Hoechst in blue. **(C)** Supernatants from organoids were collected at 3 dpi or 7 dpi and virus titers were determined by end-point dilution assay. Data is presented as Mean ± SD of two technical repetitions per datapoint of n=1 biological replicate

Figure S4. TH6744 has an antiviral effect in human iPS-cell derived cerebral organoid model.

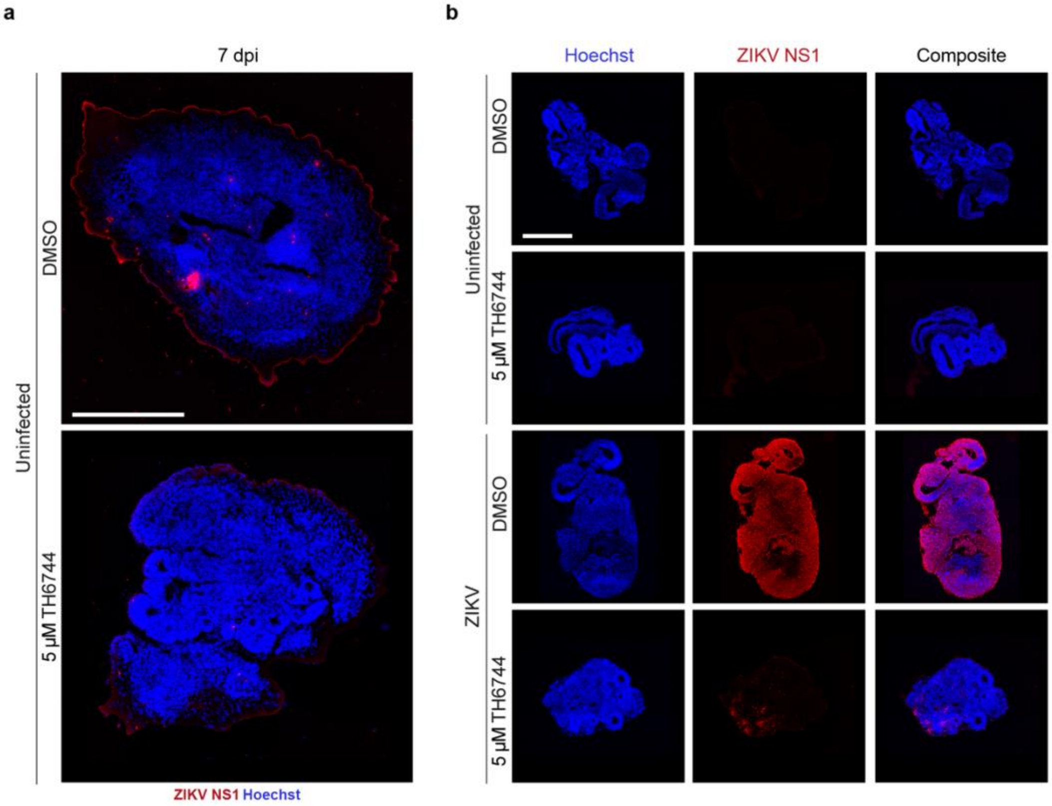

(A-B) ZIKV-infected organoids were treated with TH6744 or DMSO for 7 dpi, fixed with 4% PFA, cryosectioned and stained for Hoechst and ZIKV NS1 protein. Images were acquired by confocal microscopy. (A) Representative images of uninfected organoids from the same biological replicate as shown in Figure 3E. Scale bar equals 500 μm. ZIKV NS1 protein in red, Hoechst in blue. **(B)** Representative images of organoids from an additional biological replicate. Scale bar equals 500 μm. ZIKV NS1 protein in red, Hoechst in blue.

Figure S5. Studying ZIKV kinetics and TH6744 time-of-addition.

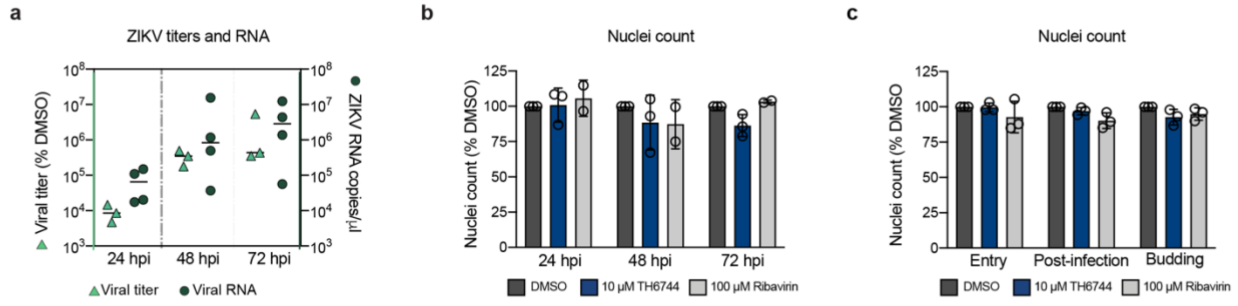

(A) U87 cells were infected with ZIKV (MOI 1) and treated with DMSO control. Viral titer (left y-axis) and extracellular vRNA (right y-axis) levels were quantified by end-point dilution assay and one-step qPCR, respectively. Raw viral titer values from Figure 4B. Data is from n=3 (viral titer) and n=4 (vRNA) biological replicates. **(B)** U87 cells were infected with ZIKV (MOI 1) and treated with indicated compounds for 24, 48 or 72 h. Nuclei count was quantified based on DAPI-positive cells and normalized to DMSO treated control at indicated time point. n=3 biological replicates. **(C)** U87 cells were infected with ZIKV (MOI 10) for a total of 24 h and were treated with DMSO control, 10 µM TH6744 or 100 µM Ribavirin for indicated periods specified in Figure 4E during early (entry), late (budding) or post-inoculation step. Nuclei count was quantified based on DAPI-positive cells and normalized to DMSO treated control at a given time point. For (B) and (C), Data is expressed as Mean ± SD of at least n=3 biological replicates and statistical significance was determined by using one-way ANOVA with Dunnett's multiple comparison analysis. $*P<0.05$, $**P<0.01$, $***P<0.001$.

Supplementary Material and methods

Chemistry

All the reagents were commercially available and were used without further purification. Analytical thin-layer chromatography was performed on silica gel 60 F-254 plates (E. Merck) and visualized with UV light. Purification of compounds by flash chromatography was achieved using a Biotage SP4 system. NMR spectra were recorded on a Bruker 400 MHz system. The chemical shifts for ^{1}H are referenced via residual solvent signal. Liquid Chromatography Mass Spectrometry (LC-MS) experiments to determine retention times (RT) and associated mass ions were performed on an Agilent MSD mass spectrometer connected to an Agilent 1100 system with: System A: Column ACE 3 C8, 3 μm, 50 x 3.0 mm maintained at 40°C. 0.1% (v/v) TFA in water (A) and MeCN (B) were used as mobile phases at a flow rate of 1 mL/min, with a gradient time of 3.0 min. System B: Column Xterra MS C18, 3.5 μm, 50 x 3.0 mm maintained at 40°C. Water (containing 10 mM NH_4HCO_3; pH = 10, A) and MeCN (B) were used as mobile phases at a flow rate of 1 mL/min, with a gradient time of 3.0 min. Preparative HPLC was performed on a Gilson HPLC system. System A: small quantities, column ACE C8, 5 μm (50 x 21.2 mm); large quantities, column ACE C8, 5 μm (150 x 30 mm); 0.1% TFA (v/v) in H2O and MeCN were used as mobile phases at a flow rate of 30 or 38 mL/min (for small and large quantity respectively) with a gradient time of 7 min. System B: small quantities, column XTerra Prep MS C18, 5 μm OBD (19 x 50 mm); large quantities, column XBridge Prep C18, 5 μm CBD (30 x 75 mm); H_2O (containing 50 mM NH_4HCO_3; pH = 10) and MeCN were used as mobile phases at a flow rate of 45 mL/min, with a gradient time of 11 min.

Synthesis of 4-(5-chloro-2-oxo-2,3-dihydro-1H-benzo[d]imidazol-1-yl)-N-(4-iodophenyl)piperidine-1-carboxamide TH3289

A mixture of 5-chloro-1-(4-piperidyl)-1H-benzimidazol-2-one (30 mg, 0.12 mmol) and 4-iodophenyl isocyanate (29 mg, 0.12 mmol) was stirred in DCM (3 mL) at r.t. for 3 h. The precipitate was collected by filtration and was washed sequentially with DCM, water, and methanol to give the title compound as a white solid (45 mg, 76%). LCMS $[M+H]^+$ 497; ^{1}H NMR (400 MHz, DMSO-d_6) δ ppm 11.05 (br. s., 1 H) 8.69 (s, 1 H) 7.52 - 7.59 (m, 2 H) 7.32 - 7.39 (m, 2 H) 7.24 (d, *J* = 8.37 Hz, 1 H) 6.97 - 7.06 (m, 2 H) 4.33 - 4.44 (m, 1 H) 4.23 - 4.33 (m, 2 H) 2.86 - 2.99 (m, 2 H) 2.15 - 2.31 (m, 2 H) 1.67 - 1.80 (m, 2 H); ^{13}C NMR (100 MHz, DMSO-d_6) δ ppm 154.4, 153.6, 140.7, 136.9, 129.5, 128.3, 124.8, 121.7, 120.1, 109.7, 108.7, 84.6, 50.3, 43.4, 28.6.

Synthesis of ethyl 1-(1-(1-((4-iodophenyl)carbamoyl)piperidin-4-yl)-2-oxo-2,3-dihydro-1H-benzo[d]imidazol-4-yl)-1H-pyrazole-4-carboxylate TH6744

Step 1: ethyl 1-(3-fluoro-2-nitrophenyl)-1H-pyrazole-4-carboxylate

A mixture of ethyl 1H-pyrazole-4-carboxylate (1.40 g, 10.0 mmol) and sodium hydride (0.440 g, 11.0 mmol; as a 60% dispersion in mineral oil) was stirred in THF (20 mL) at r.t. for 20 min. To the mixture was then added 1,3-difluoro-2-nitrobenzene (1.59 g, 10.0 mmol) and the temperature was raised to 110°C and stirring continued for 16 h. Purification by silica gel flash chromatography afforded ethyl 1-(3-fluoro-2-nitrophenyl)-1H-pyrazole-4-carboxylate (1.50 g, 54%). LCMS $[M+H]^+$ 280. ^{1}H NMR (400 MHz, $CDCl_3$) δ ppm 8.19 (s, 1 H), 8.03 (s, 1 H), 7.56 (app. td, *J* = 8.4, 5.5 Hz, 1 H), 7.32 (dt, *J* = 8.3, 1.2 Hz, 1 H), 7.28 (app. td, *J* = 8.7, 1.2 Hz, 1 H), 7.13 (q, *J* = 7.1 Hz, 2 H), 1.30 (t, *J* = 7.1 Hz, 3 H).

Step 2: tert-butyl 4-((3-(4-(ethoxycarbonyl)-1H-pyrazol-1-yl)-2-nitrophenyl)amino)piperidine-1-carboxylate

A mixture of ethyl 1-(3-fluoro-2-nitro-phenyl)pyrazole-4-carboxylate (1.00 g, 3.58 mmol), tert-butyl 4-aminopiperidine-1-carboxylate (0.789 g, 3.94 mmol) and N,N-diisopropylethylamine (0.686 mL, 3.94 mmol) was stirred in 2-propanol (10 mL) at 120 °C for 16 h. Purification by silica gel flash chromatography afforded tert-butyl 4-((3-(4-(ethoxycarbonyl)-1H-pyrazol-1-yl)-2-nitrophenyl)amino)piperidine-1-carboxylate (1.65 g, 87%). LCMS $[M+H]^+$ 460. ^{1}H NMR (400 MHz, $CDCl_3$) δ ppm 8.19 (d, *J* = 0.4 Hz, 1 H), 8.08 (d, *J* = 0.4 Hz, 1 H), 7.43 (dd, *J* = 8.7, 7.7 Hz, 1 H), 6.95 (br. d, *J* = 8.3 Hz, 1 H), 6.73 (dd, *J* = 7.7, 1.0 Hz, 1 H), 4.35 (q, *J* = 7.1 Hz, 2 H), 4.01 – 4.08 (m, 2 H), 3.59 – 3.67 (m, 1 H), 2.99 – 3.07 (m, 2 H), 2.02 – 2.09 (m, 2 H), 1.47 – 1.56 (overlapping m, 2 H), 1.49 (s, 9 H), 1.39 (t, *J* = 7.1 Hz, 3 H).

Step 3: tert-butyl 4-(4-(4-(ethoxycarbonyl)-1H-pyrazol-1-yl)-2-oxo-2,3-dihydro-1H-benzo[d]imidazol-1-yl)piperidine-1-carboxylate

To a stirred mixture of tert-butyl 4-[3-(4-ethoxycarbonylpyrazol-1-yl)-2-nitro-anilino]piperidine-1-carboxylate (1.41 g, 3.07 mmol) and $NiCl_2$ (0.080 g, 0.61 mmol) in acetonitrile/water (25 mL, 9:1) at r.t. was slowly added $NaBH_4$ (0.464 g, 12.3 mmol). The mixture was stirred for 20 min and then transferred to an extraction funnel and extracted (DCM/sat. $NaHCO_3$), dried over $MgSO_4$ and filtered. To the collected organic layer was then added bis(trichloromethyl) carbonate (0.317 g, 1.07 mmol) followed by N,N-diisopropylethylamine (1.34 mL, 7.66 mmol) and the resulting solution was stirred at r.t. for 10 min. Purification by silica gel flash chromatography afforded tert-butyl 4-(4-(4-(ethoxycarbonyl)-1H-pyrazol-1-yl)-2-oxo-2,3-dihydro-1H-benzo[d]imidazol-1-yl)piperidine-1-carboxylate (0.610 g, 44%). LCMS $[M+H]^+$ 456. ^{1}H NMR (400 MHz, DMSO-d_6) δ ppm 10.86 (s., 1 H), 8.91 (d, *J* = 0.5 Hz, 1 H), 8.13 (d, *J* = 0.5 Hz, 1 H), 7.41 (dd, *J* = 8.2, 0.5 Hz, 1 H), 7.30 (br. d, *J* = 7.8 Hz, 1 H), 7.13 (t, *J* = 8.1 Hz, 1 H), 4.40 (tt, *J* = 12.1, 3.9 Hz, 1 H), 4.29 (q, *J* = 7.1 Hz, 2 H), 4.05 – 4.18 (m, 2 H), 2.80 – 2.99 (m, 2 H), 2.18 – 2.30 (m, 2 H), 1.69 – 1.77 (m, 2 H), 1.45 (s, 9 H), 1.32 (t, *J* = 7.1 Hz, 3 H). ^{13}C NMR (100 MHz, DMSO-d_6) δ ppm 162.5, 154.3, 153.9, 142.0, 133.2, 131.7, 122.6, 121.4, 121.1, 116.1, 114.0, 108.3, 79.3, 60.4, 50.8, 43.1 (br. s), 28.9 (br. s), 28.6, 14.8.

Step 4: ethyl 1-(2-oxo-1-(piperidin-4-yl)-2,3-dihydro-1H-benzo[d]imidazol-4-yl)-1H-pyrazole-4-carboxylate

A solution of tert-butyl 4-(4-(4-(ethoxycarbonyl)-1H-pyrazol-1-yl)-2-oxo-2,3-dihydro-1H-benzo[d]imidazol-1-yl)piperidine-1-carboxylate (0.384 g, 0.842 mmol) in trifluoroacetic acid (2 mL) was stirred at r.t. for 10 min and then concentrated to give ethyl 1-(2-oxo-1-(piperidin-4-yl)-2,3-dihydro-1H-benzo[d]imidazol-4-yl)-1H-pyrazole-4-carboxylate as a trifluoroacetate salt (0.390 g, quant.). LCMS $[M+H]^+$ 356. ^{1}H NMR (400 MHz, DMSO-*d_6*) d ppm 10.95 (s, 1 H), 8.94 (s, 1 H), 8.69 (br. s., 1 H), 8.46 (br. s., 1 H), 8.15 (s, 1 H), 7.46 (d, *J* = 7.9 Hz, 1 H), 7.39 (d, *J* = 7.6 Hz, 1 H), 7.16 - 7.23 (m, 1 H), 4.54 - 4.64 (m, 1 H), 4.29 (q, *J* = 7.0 Hz, 2 H), 3.42 - 3.51 (m, 2 H), 3.04 - 3.18 (m, 2 H), 2.54 - 2.69 (m, 2 H), 1.86 - 1.97 (m, 2 H), 1.32 (t, *J* = 7.0 Hz, 3 H).

Step 5: ethyl 1-(1-(1-((4-iodophenyl)carbamoyl)piperidin-4-yl)-2-oxo-2,3-dihydro-1H-benzo[d]imidazol-4-yl)-1H-pyrazole-4-carboxylate (TH6744)

To a stirred mixture of ethyl 1-(2-oxo-1-(piperidin-4-yl)-2,3-dihydro-1H-benzo[d]imidazol-4-yl)-1H-pyrazole-4-carboxylate trifluoroacetate (0.300 g, 0.639 mmol) and N,N-diisopropylethylamine (0.223 mL, 1.28 mmol) in dichloromethane (30 mL) was added a solution of 4-iodophenyl isocyanate (0.157 g, 0.639 mmol) in dichloromethane (2 mL). The mixture was stirred at r.t. for 2 h and the solid material was collected

by filtration and washed with water followed by dichloromethane to afford the title compound as a white solid (0.300 g, 78%). LCMS $[M+H]^+$ 601; ^{1}H NMR (400 MHz, DMSO-d_6) δ ppm 10.88 (br. s., 1 H) 8.92 (s, 1 H) 8.71 (s, 1 H) 8.13 (s, 1 H) 7.54 - 7.60 (m, 2 H) 7.29 - 7.44 (m, 4 H) 7.13 (t, *J* = 8.13 Hz, 1 H) 4.40 - 4.56 (m, 1 H) 4.23 - 4.35 (m, 4 H) 2.85 - 3.06 (m, 2 H) 2.21 - 2.42 (m, 2 H) 1.71 - 1.85 (m, 2 H) 1.31 (t, *J* = 7.11 Hz, 3 H); ^{13}C NMR (100 MHz, DMSO-d_6) δ ppm 162.1, 154.4, 153.5, 141.6, 140.7, 136.9, 132.7, 131.3, 122.1, 121.7, 120.9, 120.6, 115.6, 113.5, 107.8, 84.5, 60.0, 50.5, 43.5, 28.6, 14.3.

Synthesis of N-(4-iodophenyl)-4-[2-oxo-5-(trifluoromethyl)-2,3-dihydro-1H-1,3-benzodiazol-1-yl]piperidine-1-carboxamide TH5264

Step 1: tert-butyl 4-[2-nitro-4-(trifluoromethyl)anilino]piperidine-1-carboxylate. A mixture of 1-fluoro-2-nitro-4-(trifluoromethyl)benzene (0.418 g, 2.00 mmol), tert-butyl 4-aminopiperidine-1-carboxylate (0.401 g, 2.00 mmol) and N,N-diisopropylethylamine (0.350 mL, 2.00 mmol) was stirred in 2-propanol (5 mL) at 120 °C for 12 h. The solvent was removed and the mixture was purified by silica gel flash chromatography which afforded tert-butyl 4-[2-nitro-4-(trifluoromethyl)anilino]piperidine-1-carboxylate (0.746 g, 96%). LCMS $[M]^-$ 388. ^{1}H NMR (400 MHz, $CDCl_3$) δ ppm 8.42 (br d, *J* = 1.2 Hz, 1 H), 8.24 (br d, *J* = 7.3 Hz, 1 H), 7.54 (dd, *J* = 9.1, 2.1 Hz, 1 H), 6.89 (d, *J* = 9.1 Hz, 1 H), 3.93 – 4.03 (m, 2 H), 3.60 – 3.69 (m, 1 H), 2.94 – 3.04 (m, 2 H), 1.96 – 2.04 (m, 2 H), 1.48 – 1.57 (m, 2 H), 1.41 (s, 9 H).

Step 2: tert-butyl 4-[2-oxo-5-(trifluoromethyl)-3H-benzimidazol-1-yl]piperidine-1-carboxylate. A mixture of tert-butyl 4-[2-nitro-4-(trifluoromethyl)anilino]piperidine-1-carboxylate (0.746 g, 1.92 mmol) and Pd/C (0.075 g, 10 wt%) was stirred in THF under hydrogen atmosphere at r.t. for 16 h. Thereafter N,N-diisopropylethylamine (0.670 mL, 3.85 mmol) and bis(trichloromethyl) carbonate (0.193 g, 0.671 mmol) were added. The resulting mixture was stirred at r.t. for 1 h after which it was concentrated and purified by silica gel chromatography which afforded tert-butyl 4-[2-oxo-5-(trifluoromethyl)-3H-benzimidazol-1-yl]piperidine-1-carboxylate (0.688 g, 93%). LCMS $[M\text{-isobutene}]^+$ 330. ^{1}H NMR (400 MHz, $CDCl_3$) δ ppm 9.30 (br s, 1 H), 7.34 – 7.37 (m, 2 H), 7.20 (d, *J* = 8.8 Hz, 1 H), 4.49 (tt, *J* = 12.5, 4.2 Hz, 1 H), 4.31 – 4.38 (m, 2 H), 2.84 – 2.93 (m, 2 H), 2.26 – 2.38 (m, 2 H), 1.82 – 1.88 (m, 2 H), 1.51 (s, 9H).

Step 3: 3-(4-piperidyl)-6-(trifluoromethyl)-1H-benzimidazol-2-one trifluoroacetate. A mixture of tert-butyl 4-[2-oxo-5-(trifluoromethyl)-3H-benzimidazol-1-yl]piperidine-1-carboxylate (0.688 g, 1.79 mmol) was stirred in TFA (5 mL) at r.t. for 10 min. The solvent was evaporated and traces of TFA was removed by co-evaporation with 2-propanol. No further purification was done. Yield 0.509 g (quant.). LCMS $[M+H]^+$ 286. ^{1}H NMR (400 MHz, DMSO-d6) δ ppm 11.33 (s, 1 H), 8.60 – 8.70 (m, 1 H), 8.36 – 8.48 (m, 1 H), 7.42 – 7.50 (m, 2 H), 7.26 (br. s, 1 H), 4.58 (tt, *J* = 12.2, 4.1 Hz, 1 H), 3.41 – 3.49 (m, 2 H), 3.05 – 3.17 (m, 2 H), 2.51 – 2.61 (overlapping m, 2 H), 1.86 – 1.95 (m, 2 H).

Step 4: N-(4-iodophenyl)-4-[2-oxo-5-(trifluoromethyl)-2,3-dihydro-1H-1,3-benzodiazol-1-yl]piperidine-1-carboxamide. To a stirred mixture of 3-(4-piperidyl)-6-(trifluoromethyl)-1H-benzimidazol-2-one trifluoroacetate (0.0400 g, 0.100 mmol) and N,N-diisopropylethylamine (0.175 mL, 0.100 mmol) in dichloromethane (3 mL) was added a solution of 4-iodophenyl isocyanate (0.245 g, 0.100 mmol) in dichloromethane (2 mL). The mixture was stirred at r.t. for 2 h and the solid material was collected by filtration and washed with water followed by dichloromethane to afford the title compound as a white solid (0.030 g 56%). LCMS $[M+H]^+$ 531. ^{1}H NMR (400 MHz, DMSO-d6) δ ppm 11.28 (br s, 1 H), 8.71 (s, 1 H), 7.55 – 7.59 (m, 2 H), 7.44 (br d, *J* = 8.4 Hz, 1 H), 7.34 – 7.39 (m, 3 H), 7.23 (d, *J* = 1.4 Hz, 1 H), 4.46 (tt, *J* = 12.1, 4.0 Hz, 1 H), 4.27 – 4.34 (m, 2 H), 2.90 – 3.00 (m, 2 H), 2.22 – 2.34 (m, 2 H), 1.73 – 1.80 (m, 2 H). ^{13}C NMR (100

MHz, DMSO-d_6) δ ppm 154.9, 154.4, 141.1, 137.4, 132.9, 129.1, 125.3 (q, *J* = 271 Hz), 122.2, 121.6 (q, *J* = 32 Hz), 118.2 (q, *J* = 3.9 Hz), 109.1, 105.7 (q, *J* = 3.9 Hz), 85.0, 51.0, 43.9, 29.1.

Table S1: qPCR primers

qPCR	Target	Sequence 5′ to 3′
Cell	hbActin	Unknown, used commercial primers:<br>PrimePCR™ PreAmp for Probe Assay: ACTB, Human (biorad)
Cell	Zika virus envelope gene_TaqMan probe (FAM-MGB(NFQ))	CACAAGGTGAAGCCTAC
Cell	Zika virus envelope gene_FW	ATATCGGACATGGCYTCGGA
Cell	Zika virus envelope gene_RV1	CATATTGAGTGTCTGAYTGCTTGTCA
Cell	Zika virus envelope gene_RV2	CATATTGGGTGTCTGAYTGCTTATCA
Supernatant	ZIKV 1086 [1]	CCGCTGCCCAACACAAG
Supernatant	ZIKV 1162c	CCACTAACGTTCTTTTGCAGACAT
Supernatant	ZIKV 1107_Probe	FAM-AGCCTACCTTGACAAGCAGTCAGACACTCAA-BHQ1

Table S2: Antibodies

**Target**	**Host**	**Company**	**Cat. Number**	**Dilution**
ZIKV NS1	Mouse	Abcam	ab218546	1:2000
GM130	Mouse			1:400
KDEL	Rat	Abcam	ab50601	1:400
Donkey anti-Mouse IgG (H+L) Highly Cross-Adsorbed Secondary Antibody, Alexa Fluor 488	Donkey	ThermoFisher Scientific	A-21202	1:800
Goat anti-Mouse IgG (H+L) Cross-Adsorbed Secondary Antibody, Alexa Fluor 647	Goat	ThermoFisher Scientific	A-21235	1:800
Goat anti-Rabbit IgG (H+L) Highly Cross-Adsorbed Secondary Antibody, Alexa Fluor 488	Goat	ThermoFisher Scientific	A11008	1:800
Goat anti-Rat IgG (H+L) Cross-Adsorbed Secondary Antibody, Alexa Fluor 555	Goat	ThermoFisher Scientific	A21434	1:800

Table S3: DNA Dyes

Dye	Company	Cat Number	Dilution
DAPI (4',6-Diamidino-2-Phenylindole, Dihydrochloride)	ThermoFisher Scientific	D1306	1:1000
Hoechst 33342	Eugene	H3570	1:1000

Supplementary reference

1. Lanciotti, R. S. *et al.* Genetic and serologic properties of Zika virus associated with an epidemic, Yap State, Micronesia, 2007. *Emerg. Infect. Dis.* **14**, 1232–9 (2008).
